# The Tail and the Dog

**Published:** 1906-06

**Authors:** 


### Abstracts and Selections.

### The Tail and the Dog.

[The following editorial appeared in the May number of *The Southern Practitioner,* Nashville, Tenn. It was written by the veteran editor, Professor Deering J. Roberts, than whom there does not live a more honorable, just, generous and esteemed physician in the world. Professor Roberts is known to be one of the sticklers for professional ethics, as are all Southern physicians of the old regime, at least; and having been a teacher of medicine more than a quarter of a century, he has instilled high ideals into the minds of his pupils. He was for many years professor of medicine in the University of Nashville, and now holds that chair in the Medical Department of the University of the South, at Sewanee, Tenn. He is ex-president of the Tennessee State Medical Association and ex-surgeon C. S. A. *The Southern Practitioner* is now in its *twenty-eighth year* under the editorial management of this able, distinguished and universally honored and esteemed physician; he is the *dean* of the medical press. As such his words carry weight and are entitled to respect. I endorse his views on the subject treated. *Fiat justitia ruat caelum.*]

